# Loss of mitochondrial ATPase ATAD3A contributes to nonalcoholic fatty liver disease through accumulation of lipids and damaged mitochondria

**DOI:** 10.1016/j.jbc.2022.102008

**Published:** 2022-05-02

**Authors:** Liting Chen, Yuchang Li, Chantal Sottas, Anthoula Lazaris, Stephanie K. Petrillo, Peter Metrakos, Lu Li, Yuji Ishida, Takeshi Saito, Samuel Garza, Vassilios Papadopoulos

**Affiliations:** 1 Department of Pharmacology and Pharmaceutical Sciences, School of Pharmacy, University of Southern California, Los Angeles, California, USA; 2 Research Institute of the McGill University Health Center, Montreal, Quebec, Canada; 3 Department of Surgery, McGill University, Montreal, Quebec, Canada; 4 Department of Medicine, Division of Gastrointestinal and Liver Diseases, Keck School of Medicine, University of Southern California, Los Angeles, California, USA; 5 Research & Development Department, PhoenixBio, Co, Ltd, Higashi-Hiroshima, Hiroshima, Japan; 6 University of Southern California Research Center for Liver Diseases, Los Angeles, California, USA

**Keywords:** ATAD3A, NAFLD, cholesterol, triglyceride, free fatty acid, fatty acid oxidation, autophagy, mitophagy, mitochondrial respiration, 3-MA, 3-methyladenine, ATAD3A, ATPase Family AAA Domain Containing 3A, BSA, bovine serum albumin, CPT1A, carnitine palmitoyltransferase 1A, ER, endoplasmic reticulum, FAS, fatty acid synthase, FBS, fetal bovine serum, FC, free cholesterol, HLCM, humanized liver chimeric mice, HMGR, HMG-CoA reductase, KD, knockdown, LC3-II, light chain 3-II, LDs, lipid droplets, LXRA, liver X receptor alpha, MCD, methionine-choline deficient, MTT, 3-(4,5-dimethylthiazol-2-yl)-2,5-diphenyl-2H-tetrazolium bromide, NAFLD, nonalcoholic fatty liver disease, NASH, nonalcoholic steatohepatitis, PHH, primary human hepatocytes, PINK1, PTEN-induced kinase 1, PLIN2, Perilipin 2, PPARA, peroxisome proliferator activated receptor alpha, SREBP1c, sterol regulatory element-binding transcription factor 1, SS, simple steatosis, TEM, transmission electron microscopy, TG, triglyceride

## Abstract

Mitochondrial ATPase ATAD3A is essential for cholesterol transport, mitochondrial structure, and cell survival. However, the relationship between ATAD3A and nonalcoholic fatty liver disease (NAFLD) is largely unknown. In this study, we found that ATAD3A was upregulated in the progression of NAFLD in livers from rats with diet-induced nonalcoholic steatohepatitis and in human livers from patients diagnosed with NAFLD. We used CRISPR-Cas9 to delete ATAD3A in Huh7 human hepatocellular carcinoma cells and used RNAi to silence ATAD3A expression in human hepatocytes isolated from humanized liver-chimeric mice to assess the influence of ATAD3A deletion on liver cells with free cholesterol (FC) overload induced by treatment with cholesterol plus 58035, an inhibitor of acetyl-CoA acetyltransferase. Our results showed that ATAD3A KO exacerbated FC accumulation under FC overload in Huh7 cells and also that triglyceride levels were significantly increased in ATAD3A KO Huh7 cells following inhibition of lipolysis mediated by upregulation of lipid droplet-binding protein perilipin-2. Moreover, loss of ATAD3A upregulated autophagosome-associated light chain 3-II protein and p62 in Huh7 cells and fresh human hepatocytes through blockage of autophagosome degradation. Finally, we show the mitophagy mediator, PTEN-induced kinase 1, was downregulated in ATAD3A KO Huh7 cells, suggesting that ATAD3A KO inhibits mitophagy. These results also showed that loss of ATAD3A impaired mitochondrial basal respiration and ATP production in Huh7 cells under FC overload, accompanied by downregulation of mitochondrial ATP synthase. Taken together, we conclude that loss of ATAD3A promotes the progression of NAFLD through the accumulation of FC, triglyceride, and damaged mitochondria in hepatocytes.

Non-alcoholic fatty liver disease (NAFLD) is now the leading cause of chronic liver disease in the United States and the second most common indicator for liver transplantation (1). NAFLD is a condition where excess fat accumulates in the liver, inducing impaired hepatocyte function and inflammation leading to liver injury in the absence of alcohol. NAFLD is characterized by excessive triglyceride (TG) and cholesterol accumulation and constitutes a disease spectrum, with stages ranging from simple steatosis (SS) with excessive fat accumulation in the liver to nonalcoholic steatohepatitis (NASH), where inflammation and fibrosis first appear. NASH can progress further into cirrhosis and hepatocellular carcinoma at end stages (2).

Free cholesterol (FC) has been shown to disrupt mitochondrial function and induce hepatocyte death, contributing to NAFLD progression (3). In SS, excessive fats such as cholesterol and TG accumulate in hepatocytes, disrupting the intracellular homeostasis of liver cells, resulting in liver injury. Lipid disruption, mitochondrial dysfunction, endoplasmic reticulum (ER) stress, and reactive oxygen species have been shown to contribute to liver-cell damage and promote the progression of SS to NASH with concomitant fibrosis (4, 5).

Recently, it has been proposed that NAFLD is a mitochondrial disease because besides being the cell's power plants, mitochondria are also responsible for cholesterol transport, fatty acid oxidation, and generation of reactive oxygen species. Mitochondrial dysfunction renders liver cells susceptible to damage and extracellular risk factors by disrupting intracellular metabolism and signal transduction (6).

ATPase Family AAA Domain Containing 3A (ATAD3A) is a ubiquitous mitochondrial transmembrane ATPase (7, 8) and plays a role in the cholesterol transport from intracellular stores into mitochondria (7, 9, 10, 11). ATAD3A is critical for mitochondrial membrane mtDNA replication, protein synthesis, cristae structure, and mitochondrial shape (12, 13, 14, 15). Human subjects with ATAD3A- and ATAD3B-cluster deletions showed elevated FC in human fibroblasts, revealing the involvement of ATAD3A in the maintenance of cholesterol homeostasis (13). In addition, even though ATAD3A was recognized as an inner mitochondrial membrane protein, the 50 amino-acid N terminus of ATAD3 was proposed to insert into the outer mitochondrial membrane and associated organelles such as the ER (8, 10), implying potential spatial interactions with other organelles. It was reported that ATAD3A interacted with glucose regulatory protein 78, a protein located in the ER, indicating the role of ATAD3A in the regulation of ER stress (16). Furthermore, ATAD3A has been shown to promote cell survival through the regulation of the mTOR in primary cow epithelial cells (17). Although emerging evidence links ATAD3A to mitochondrial structure, function, cholesterol homeostasis, mitochondrial interactions with other cellular organelles, and cell survival, the role of ATAD3A under excess fat conditions and its relationship with NAFLD, a mitochondrial disease, have never been explored.

To gain insight into the function of ATAD3A in the progression of NAFLD, we characterized ATAD3A expression in liver samples from rats with NASH along with matching controls and liver specimens from patients with SS, NASH, cirrhosis, or normal tissue. ATAD3A was found to be upregulated with the progression of NAFLD, revealing the possible involvement of ATAD3A. Moreover, loss of ATAD3A increased FC and TG accumulation under FC overload in Huh7 cells and blocked autophagosome degradation in both Huh7 cells and fresh human hepatocytes isolated from humanized liver chimeric mice (HLCM). Mitophagy was largely inhibited after the loss of ATAD3A under FC overload in Huh7 cells. ATAD3A KO Huh7 cells under FC overload also showed reduced mitochondrial respiration. Collectively, our results indicated that ATAD3A plays an essential role in the progression of NAFLD.

## Results

### ATAD3A levels are increased in rats with NASH phenotype and humans with NAFLD

**Figure 1:**
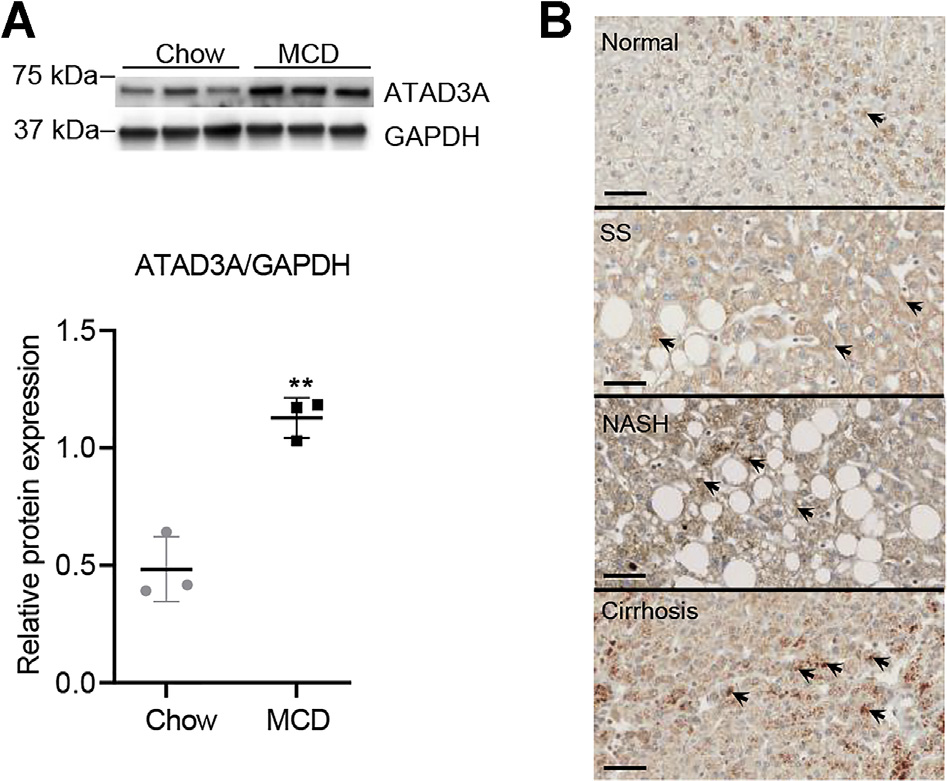
**ATAD3A was upregulated in rats with NASH and human patients with NAFLD.***A*, immunoblots for ATAD3A in rats fed with chow or MCD diet for 8 weeks (*top left*); quantification using ImageJ (*bottom left*). Data are presented as mean ± SD. ∗∗*p* < 0.01 by unpaired two-tailed student's t test. *B*, immunohistochemistry for ATAD3A in normal human tissue or patients with SS, NASH, or cirrhosis. A detailed description of the human liver samples used is summarized in Table S1. *Black arrows*, ATAD3A antibody-stained area. The scale bar represents 50 μm. MCD, methionine-choline deficient; NASH, nonalcoholic steatohepatitis; SS, simple steatosis.

To assess the expression levels of ATAD3A in the progression of NAFLD, we fed Sprague Dawley rats a methionine-choline deficient (MCD) diet for 8 weeks to induce a NASH phenotype (18). Chow-fed rats were used as controls. Rat livers were isolated and subjected to immunoblot analysis to assess the levels of ATAD3A. Immunoblots and corresponding quantification showed that ATAD3A expression was significantly upregulated in livers of rats fed the MCD diet compared to the chow diet (Fig. 1*A*). To see if this is applied to humans, we stained liver sections from normal individuals and patients with SS, NASH, or cirrhosis with anti-ATAD3A antibody. The human liver samples were first blind-scored using the NAFLD activity score system by a pathologist, regardless of gender. Then, 12 samples were randomly picked as representative of different liver stages by NAS grades of normal, steatosis, NASH, and cirrhosis (n = 3 for each stage) for further analysis. The results showed that in patients with SS, NASH, and cirrhosis, the expression of ATAD3A in the liver was increased compared to normal (Fig. 1*B*). Collectively, this suggests that ATAD3A may either play a role in the origin of NAFLD or be part of the disease phenotype.

### Characterization of ATAD3A KO in Huh7 cells

**Figure 2:**
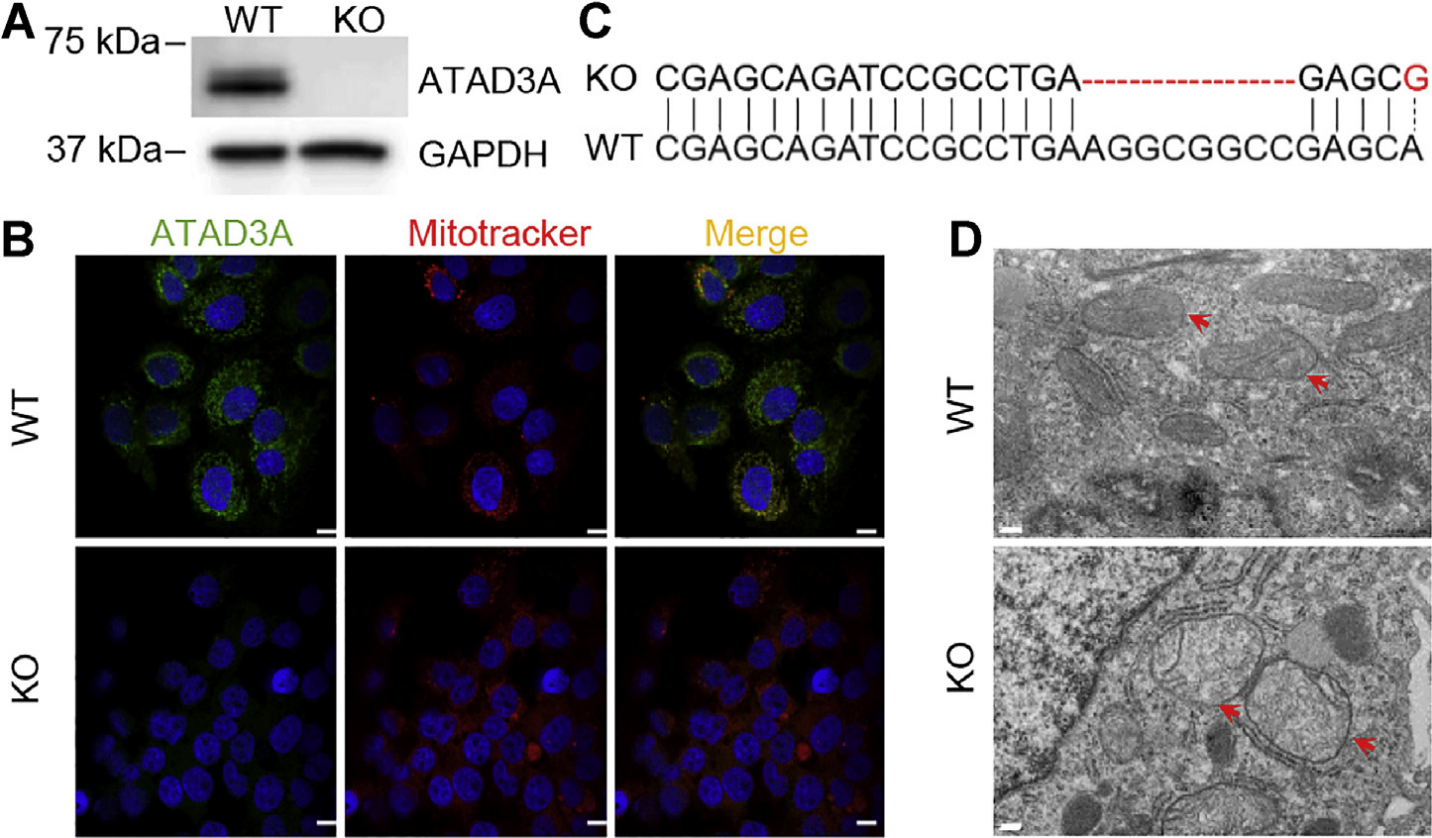
**Characterization of ATAD3A KO in Huh7.***A*, immunoblots for ATAD3A in Huh7 WT and ATAD3A KO cells. *B* confocal images for ATAD3A (*green*) and MitoTracker (*red*) in Huh7 WT and ATAD3A KO cells. The scale bar represents 20 μm. *C*, next-generation sequencing for ATAD3A identified the deletion of eight base pairs and one base pair mutation at exon 7 of ATAD3A. *D*, mitochondrial morphology in Huh7 WT and ATAD3A KO cells under TEM. *Red arrows*, mitochondria. The scale bar represents 0.2 μm. TEM, transmission electron microscopy.

To examine the role of ATAD3A in the progression of NAFLD, CRISPR-Cas9 was performed to generate an ATAD3A KO Huh7 cell line. The loss of ATAD3A was demonstrated by immunoblot analysis (Fig. 2*A*).

Confocal microscopy imaging demonstrated that most of ATAD3A colocalized with the mitochondrial marker MitoTracker in WT Huh7 cells. Image analysis showed a Pearson’s coefficient of 0.937 and a Mander’s coefficient of 0.87 (fraction of ATAD3A overlapping with MitoTracker). ATAD3A fluorescence was not seen in the ATAD3A KO Huh7 cells, confirming the loss of ATAD3A (Fig. 2*B*).

Next-generation sequencing identified a deletion of 8 base pairs and a mutation in 1 base pair in ATAD3A genomic DNA at the sgRNA-targeted sequence in exon 7 (Fig. 2*C*). Notably, transmission electron microscopy (TEM) images revealed a rounded mitochondrial structure and disrupted mitochondrial cristae in the ATAD3A KO Huh7 cells (Fig. 2*D*), which is consistent with the mitochondrial cristae alteration in MA-10 cells in response to ATAD3A siRNA (19). Collectively, these results suggested that we successfully established an ATAD3A KO Huh7 cell line.

### ATAD3A KO exacerbates FC accumulation under FC overload

**Figure 3:**
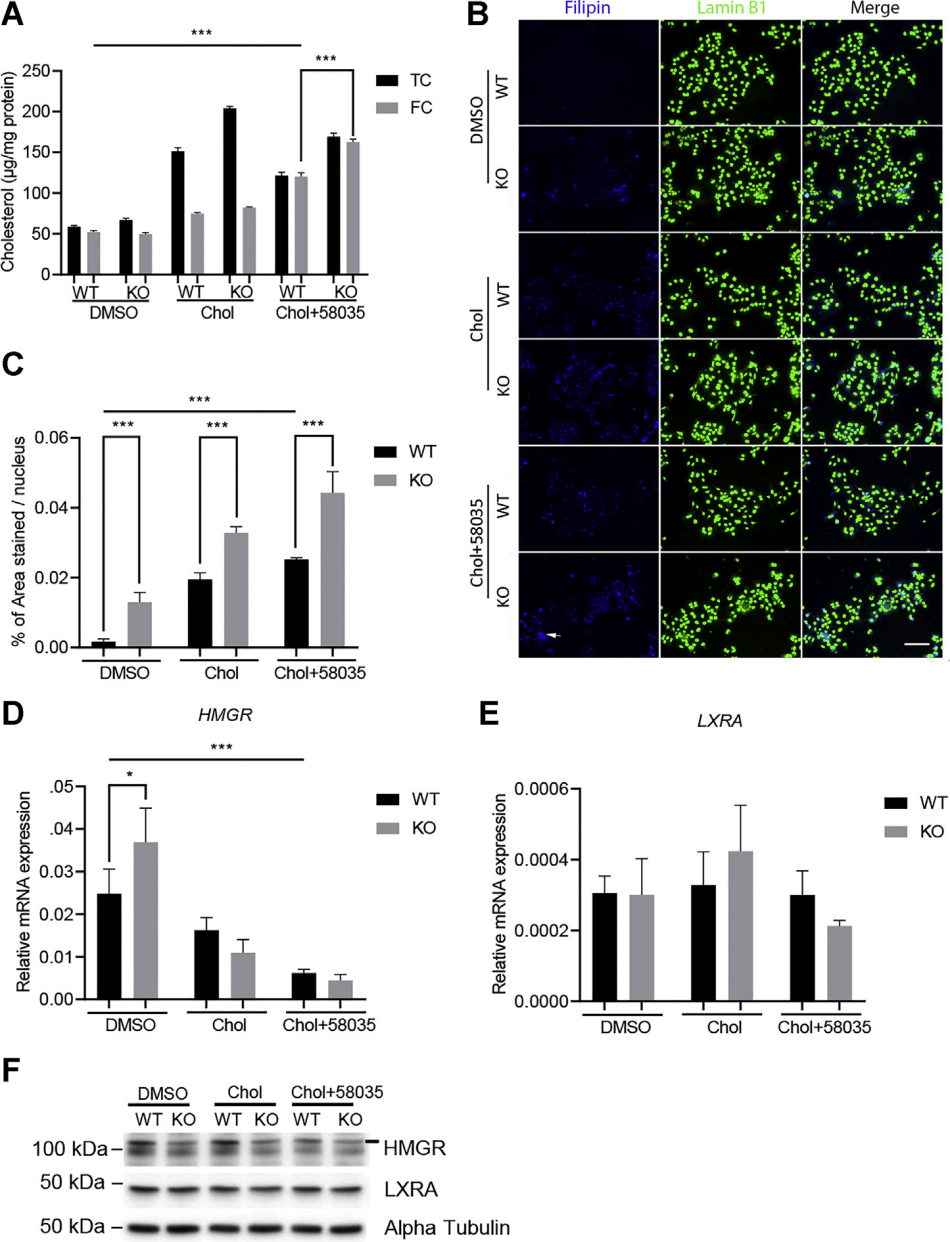
**ATAD3A KO exacerbated FC accumulation under FC overload in Huh7 cells.***A*, cholesterol quantification of ATAD3A KO and WT cells treated with DMSO, cholesterol (Chol), or Chol plus 58035, an acetyl-CoA acetyltransferase inhibitor, for 24 h (n = 3). *B*, WT or ATAD3A KO Huh7 cells were treated as indicated for 24 h after overnight incubation with DMEM supplemented with 10% of lipoprotein-deficient serum. Cells were fixed and stained with filipin (*blue*) and Lamin B1 (*green*) after incubation. *White arrows*, large puncta stained with filipin. The scale bar represents 130 μm. *C*, quantification for B. Percentage of area stained with filipin/nucleus for four images of WT or ATAD3A KO Huh7 cells treated as indicated analyzed with ImageJ. *D* and *E*, qPCR for HMGR and LXRA mRNA expression normalized to S18 from ATAD3A KO and Huh7 WT cells (n = 3). *F*, immunoblot analyses for HMGR and LXRA in WT or ATAD3A KO Huh7 cells treated as indicated. Data are presented as mean ± SD. ∗∗∗*p* < 0.001, ∗*p* < 0.05 by two-way ANOVA for cholesterol quantification and one-way ANOVA for Filipin quantification and qPCR. FC, free cholesterol; HMGR, 3-hydroxy-3-methylglutaryl-CoA reductase; LXRA, liver X receptor alpha; qPCR, quantitative PCR; TC, total cholesterol.

Since FC rather than total cholesterol contributes to the progression of NAFLD (3), we induced FC overload in Huh7 cells by treatment with cholesterol and 58035, an inhibitor of acetyl-CoA acetyltransferase 2, a liver-specific enzyme responsible for the conversion of FC to cholesterol ester (3). After treatment of cholesterol plus 58035, FC levels were significantly increased in both WT and KO cells, indicating that the treatment successfully induced FC overload in Huh7 cells (Fig. 3*A*). Interestingly, FC levels in KO cells were significantly higher than WT under FC overload (Fig. 3*A*). Treated Huh7 cells were also stained with Filipin, a dye commonly used to stain FC, and observed under fluorescent microscopy. As shown in Figure 3*B*, the blue Filipin puncta were more abundant and larger in KO cells than WT under FC overload, as well as control conditions. The Filipin positive signals were then quantified and normalized to the number of nuclei. As shown in Figure 3*C*, the % area stained with Filipin per cell was significantly higher in KO cells than WT under FC overload and control conditions.

To examine the mechanism underlying ATAD3A KO deteriorated FC accumulation under FC overload, we next tested whether cholesterol synthesis or metabolism was altered after the loss of ATAD3A. The quantitative PCR (qPCR) results did not show obvious differences in mRNA levels of HMG-CoA reductase (HMGR), the rate-limiting enzyme for cholesterol synthesis, between KO and WT under FC overload, even though ATAD3A KO significantly upregulated mRNA expression of HMGR in controls (Fig. 3*D*). HMGR protein levels were suppressed after loss of ATAD3A under control conditions, but the difference between WT and ATAD3A KO cells was less obvious under FC overload (Fig. 3*F*). ATAD3A KO tended to suppress mRNA expression of liver X receptor alpha (LXRA), a gene involved in cholesterol metabolism, but protein levels remained stable under FC overload (Fig. 3, *E* and *F*). Taken together, ATAD3A KO induces FC escalation upon FC accumulation without causing significant change to cholesterol synthesis and metabolism under FC overload, suggesting that ATAD3A may affect other pathways involved in cholesterol regulation, such as cholesterol secretion.

### ATAD3A KO increases TG accumulation

**Figure 4:**
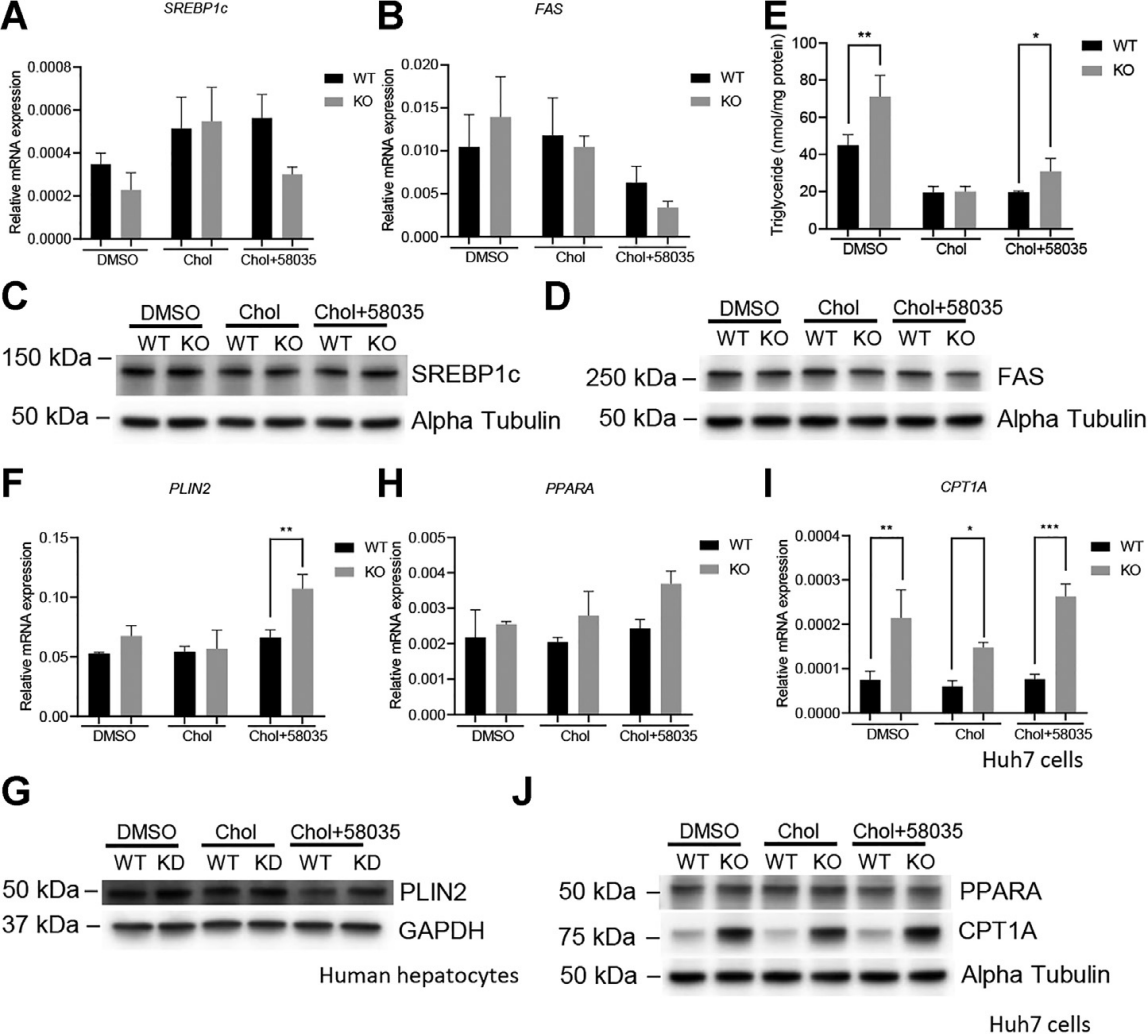
**ATAD3A KO induces TG accumulation *via* inhibited lipolysis.** Treatments are the same as in Figure 3*A* and *B*, qPCR analysis of SREBP1c, FAS mRNA expression normalized to S18 using ATAD3A KO and Huh7 WT cells treated as indicated for 24 h after overnight incubation with DMEM supplemented with 10% of lipoprotein-deficient serum (n = 3). *C* and *D*, immunoblots for SREBP1c and FAS in WT or ATAD3A KO Huh7 cells treated as indicated. Immunoblots in panel (*C*) and Figure 3*F* share the same alpha-Tubulin control because the antibodies in these figures were incubated with proteins separated on the same membrane. *E*, TG quantification for ATAD3A KO or WT Huh7 cells treated as indicated (n = 3). *F*, qPCR analysis of PLIN2 mRNA expression normalized to S18 using ATAD3A KO and Huh7 WT cells treated as indicated (n = 3). *G*, immunoblot analysis for PLIN2 protein expression in human hepatocytes subjected to ATAD3A siRNA or universal control siRNA under the treatment as indicated for 24 h. *H* and *I*, qPCR analysis of PPARA and CPT1A mRNA expression normalized to S18 using ATAD3A KO and Huh7 WT cells treated as indicated (n = 3). *J*, immunoblots for PPARA and CPT1A in WT or ATAD3A KO Huh7 cells treated as indicated. Immunoblots in panels (*D*) and (*J*) share the same alpha-Tubulin because the antibodies in (*D*) and (*J*) were incubated with proteins separated on the same membrane. Data are presented as mean ± SD. ∗∗∗*p* < 0.001, ∗∗*p* < 0.01, ∗*p* < 0.05 by one-way ANOVA. Chol, cholesterol; CPT1A, carnitine palmitoyltransferase 1A; FAS, fatty acid synthase; KD, knockdown; PLIN2, Perilipin 2; PPARA, peroxisome proliferator activated receptor a; qPCR, quantitative PCR; SREBP1c, plasminogen activator inhibitor 1 RNA-binding protein 1c; TG, triglyceride.

Next, we are interested to see if ATAD3A KO alters TG homeostasis. To this end, qPCR was performed to measure the expression of lipogenic genes in Huh7 cells, including sterol regulatory element-binding transcription factor 1 (SREBP1c) and fatty acid synthase (FAS). SREBP1c is the key transcription factor that masters the activation of genes for fatty acid synthesis, whereas FAS is the rate-limiting enzyme for fatty acid *de novo* biosynthesis (20). It was found that both SREBP1c and FAS mRNA levels were reduced to nearly 50% after the loss of ATAD3A under FC overload, not being statistically significant (Fig. 4, *A* and *B*). At the protein level, FAS was moderately downregulated in ATAD3A KO cells under FC overload, whereas no obvious change was seen for SREBP1c (Fig. 4, *C* and *D*). These data suggest the lower free fatty acids production in the ATAD3A KO cells under FC overload. Interestingly, however, ATAD3A KO significantly increased TG accumulation under FC overload and control conditions in Huh7 cells, suggesting that lipolysis may be inhibited in ATAD3A KO cells (Fig. 4*E*). Since TG is stored in lipid droplets, we stained cells with Nile red, a dye used to stain intracellular neutral lipids in lipid droplets. Image analysis showed an increased number of small lipid droplets in ATAD3A KO cells compared to WT Huh7 cells under control and FC overload conditions (Fig. S1, *A* and *B*).

Being a negative regulator for lipolysis, Perilipin 2 (PLIN2) is a protein associated with lipid droplets' (LDs) surface, stabilizing LDs and protecting neutral lipids from enzymatic metabolism. Liver-specific ablation of PLIN2 reduced neutral lipids and liver steatosis in mice fed with MCD diet, whereas PLIN2 overexpression in HepG2 cells triggered neutral lipid accumulation (21, 22). As expected, PLIN2 mRNA expression was significantly upregulated in ATAD3A KO Huh7 cells compared to WT under FC overload, indicating the inhibited lipolysis in the ATAD3A KO Huh7 cells (Fig. 4*F*). Moreover, terminally differentiated human hepatocytes were freshly isolated from the liver of HLCM and subjected to either ATAD3A siRNA to knock down ATAD3A or universal siRNA as the control followed by the same treatment for Huh7 cells. Consistently, FC overload suppressed PLIN2 protein expression, whereas ATAD3A knockdown (KD) by siRNA increased PLIN2 expression back to a normal level in human hepatocytes (Fig. 4*G*). Taken together, ATAD3A KO induces TG accumulation under FC overload as well as control conditions *via* inhibition of lipolysis.

Despite less fatty acid synthesis, as suggested by the downregulation of lipogenic gene expression following the loss of ATAD3A, ATAD3A KO significantly upregulated mRNA and protein expression of carnitine palmitoyltransferase 1A (CPT1A), the rate-limiting enzyme for fatty acid oxidation, and displayed the tendency to upregulate mRNA but not protein of peroxisome proliferator activated receptor alpha (PPARA), one of the upstream regulators of CPT1A, under FC overload in Huh7 cells, which may be the result of compensation for less free fatty acids store and lack of energy production (Fig. 4, *H*–*J*).

### ATAD3A KO blocks autophagosome degradation and induces cell death under FC overload

**Figure 5:**
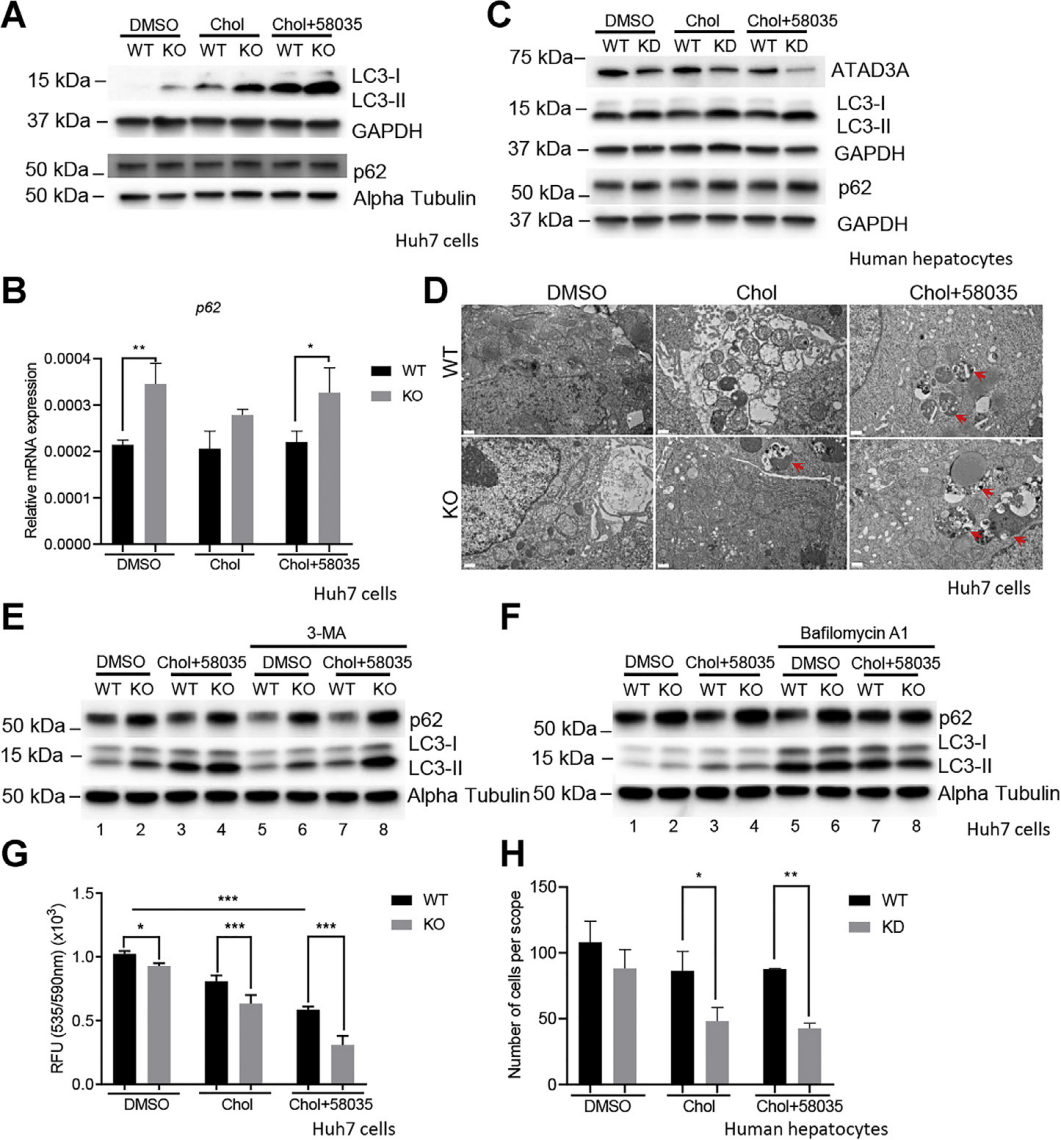
**ATAD3A KO blocks autophagy and induces cell death under FC overload in Huh7 cells and human hepatocytes.** Immunoblots for LC3-I, LC3-II, and p62 in WT or ATAD3A KO Huh7 (A), and ATAD3A, LC3-I, LC3-II, and p62 in KD human hepatocytes isolated from HLCM (C) treated as indicated for 24 h. Immunoblots in panel (C) and Figure 4*G* share the same GAPDH control because the antibodies in these figures were incubated with proteins separated on the same membrane. *B*, qPCR analysis of p62 mRNA expression normalized to S18 using ATAD3A KO and Huh7 WT cells treated as indicated (n = 3). *D*, TEM for WT or ATAD3A KO Huh7 cells treated as indicated for 24 h. *Red arrows*, autophagolysosome. The scale bar represents 0.5 μm. *E* and *F*, immunoblots for LC3-I, LC3-II, and p62 for WT or ATAD3A KO Huh7 cells pretreated with 3-MA or bafilomycin A1 for 1 h before cholesterol (Chol) or Chol plus 58035 treatment for 24 h. *G*, MTT (n = 6) for WT or ATAD3A KO Huh7 cells treated as indicated. *H*, cell count under the microscope for human hepatocytes with ATAD3A siRNA or universal control siRNA treated as indicated for 24 h (n = 3). Data are presented as mean ± SD. ∗∗∗*p* < 0.001, ∗∗*p* < 0.01, ∗*p* < 0.05 by one-way ANOVA. HLCM: humanized liver chimeric mice; FC, free cholesterol; KD, knockdown; MTT, 3-(4,5-dimethylthiazol-2-yl)-2,5-diphenyltetrazolium bromide; qPCR, quantitative PCR; RFU, relative fluorescent units; TEM, transmission electron microscopy.

Because autophagy plays an essential role in the progression of SS and is inducible by FC overload (23, 24), we next examined whether the loss of ATAD3A could influence autophagy under FC overload. As shown in Figure 5*A*, FC overload dramatically upregulated the expression of microtubule-associated protein 1A/1B-light chain 3-II (LC3-II), a wildly-used marker for autophagy, and ATAD3A KO pushed this process further. The mRNA expression levels of autophagic adapter, p62, was also significantly increased in ATAD3A KO Huh7 cells compared to WT under FC overload and control conditions despite only a slight increase in p62 protein levels following the loss of ATAD3A (Fig. 5, *A* and *B*). To further confirm these results, LC3-II and p62 protein levels were also measured in human hepatocytes with ATAD3A KD by siRNA under the same treatment conditions as Huh7 cells. Consistently, ATAD3A KD dramatically upregulated LC3-II and p62 protein levels in human hepatocytes (Fig. 5*C*). Furthermore, more abundant and larger autophagolysosomes were observed in ATAD3A KO Huh7 cells than in WT under FC overload, as shown in the TEM images (Fig. 5*D*).

As known, the upregulation of LC3-II and accumulation of autophagolysosomes can be attributed to either elevation in autophagosome formation or blockade of autophagosome degradation. To further tease out which process was affected by the loss of ATAD3A, we pretreated cells with autophagy inhibitors 3-methyladenine (3-MA), a PI3K inhibitor, or bafilomycin A1, an inhibitor of autophagosome-lysosome fusion, for 1 h before cholesterol and 58035 exposure. As shown in Figure 5*E*, 3-MA effectively reduced LC3-II levels increased by FC overload in WT cells (lane 1, 3, and 7), suggesting that FC overload induces autophagosome formation. Furthermore, the presence of bafilomycin A1 further increased LC3-II induced by FC overload in WT cells (lane 1, 3, and 7), indicating that autophagic degradation is unaffected under FC overload (Fig. 5*F*). Therefore, FC overload increases autophagic flux.

Even though 3-MA efficiently reduced LC3-II and p62 expression in WT cells under control and FC overload conditions (Fig. 5*E*, lane 1 *versus* 5 and 3 *versus* 7), the upregulation of LC3-II and p62 in ATAD3A KO cells was barely reversed by 3-MA, suggesting that ATAD3A KO does not induce autophagosome formation (Fig. 5*E*, lane 2 *versus* 6 and 4 *versus* 8). On the other hand, the upregulation of LC3-II in KO cells over WT was lost in the presence of bafilomycin A1 under FC overload or control conditions (lane 1, 2 *versus* 5, 6 and 3, 4 *versus* 7, 8), suggesting that autophagolysosomal activity was reduced and autophagosome degradation was blocked after loss of ATAD3A. In contrast, the transient overexpression of ATAD3A after FC accumulation with cholesterol and 58035 treatments enhanced the autophagic degradation evidenced by increased LC3-II and degraded p62 compared to WT (Fig. S2).

It has been reported that elevated autophagy is a protective mechanism for cells under FC overload and inhibition of autophagy enhanced cell apoptosis (24). Therefore, we speculated that loss of ATAD3A would reduce cell viability through hinderance of autophagy. As expected, the 3-(4,5-dimethylthiazol-2-yl)-2,5-diphenyl-2H-tetrazolium bromide (MTT) results showed that cell viability was significantly reduced in ATAD3A KO Huh7 cells or KD human hepatocytes compared to WT under FC overload (Fig. 5, *G* and *H*). Taken together, these data indicate that ATAD3A KO blocks autophagosome degradation under FC overload and control conditions, reducing cell survival.

### ATAD3A KO inhibits PINK1-Parkin–meditated mitophagy and mitochondrial respiration

**Figure 6:**
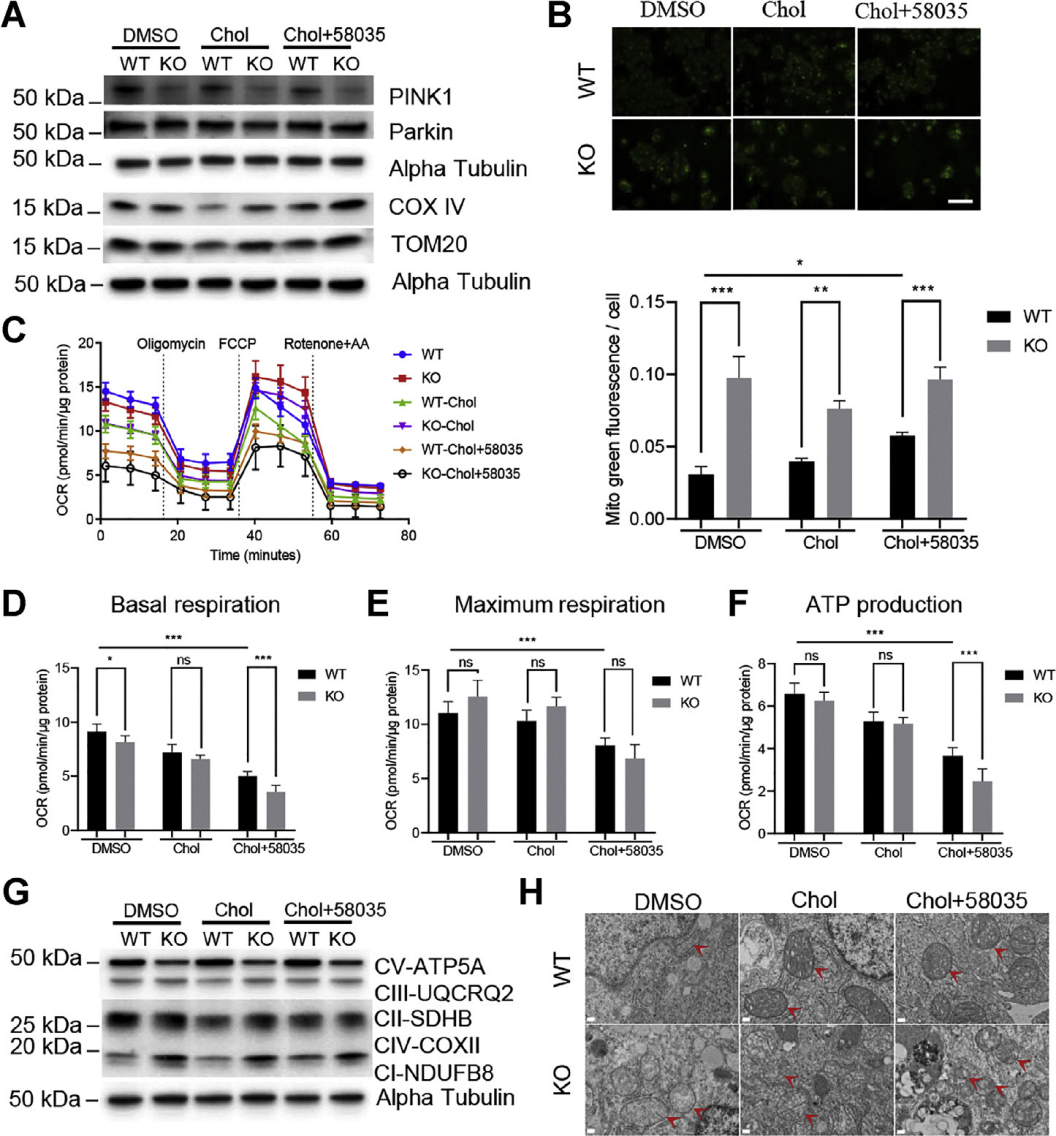
**ATAD3A KO inhibits PINK1-Parkin–dependent mitophagy and mitochondrial respiration in Huh7 cells.***A*, immunoblots for PINK1, Parkin, COX IV, and TOM20 in WT or ATAD3A KO Huh7 treated with DMSO, Chol, or Chol plus 58035 for 24 h. *B*, Mitotracker green staining (*upper*) and quantification (*lower*) for WT or ATAD3A KO Huh7 treated as indicated. The scale bar represents 130 μm. *C*, OCR Seahorse assay for Huh7 WT and ATAD3A KO cells treated as indicated. *D*–*F*, basal respiration, maximum respiration, and ATP production of Huh7 WT and ATAD3A KO cells treated as indicated. *G*, immunoblots for oxidative phosphorylation enzymes in WT or ATAD3A KO Huh7 cells treated as indicated. *H*, representative mitochondrial structure TEM images of ATAD3A KO Huh7 cells or WT cells. *Red arrows*, mitochondria. The scale bar represents 0.2 μm. Data are presented as mean ± SD. ∗∗∗*p* < 0.001, ∗∗*p* < 0.01, ∗*p* < 0.05 by one-way ANOVA. Chol, cholesterol; OCR, oxygen consumption rate; PINK1, PTEN-induced kinase 1; ROS, reactive oxygen species; ns, not significant; TEM, transmission electron microscopy.

Since ATAD3A is located in inner mitochondrial membrane, we examined whether mitophagy, a selective autophagy process of mitochondria, was affected after the loss of ATAD3A under FC overload. As shown in Figure 6*A*, the expression of PTEN-induced kinase 1 (PINK1), the initial adapter for PINK1-Parkin mediated mitophagy, was dramatically downregulated after the loss of ATAD3A under FC overload and basal conditions, suggesting that ATAD3A is essential for the PINK1-Parkin–mediated mitophagy and loss of ATAD3A will inhibit mitophagy. Consistently, MitoTracker green, an indicator of mitochondrial mass, was significantly increased after the loss of ATAD3A under both control conditions and FC overload, suggesting accumulation of mitochondria after deletion of ATAD3A (Fig. 6*B*). The upregulation of mitochondrial proteins cytochrome c oxidase subunit 4 (COX IV) and translocase of outer mitochondrial membrane 20 (TOM20) further confirmed the increased mitochondrial mass in ATAD3A KO cells under FC overload (Fig. 6*A*).

The analysis of mitochondrial function using a Seahorse analyzer was undertaken to see whether the accumulation of mitochondria after ATAD3A deletion is beneficial to the energy supply of cells. The results showed that basal respiration and ATP production significantly dropped after the loss of ATAD3A under FC overload, even though only a decline trend was seen for maximum respiration, suggesting that ATAD3A KO tends to accumulate dysfunctional mitochondria in cells under FC overload (Fig. 6, *C*–*F*).

To examine whether the compromised mitochondrial respiration and ATP production after loss of ATAD3A were linked to the mitochondrial oxidative phosphorylation complex, the expression of five oxidative phosphorylation complex enzymes was measured. As shown in Figure 6*G*, although ATAD3A KO did not affect complex II and complex III expression, complex IV and complex I were obviously upregulated in ATAD3A KO cells compared to WT under both control conditions and FC overload. Intriguingly, however, complex IV, the ATP synthase responsible for ATP generation, was drastically downregulated in ATAD3A KO cells compared to WT under control conditions and FC overload. These data indicate that ATAD3A is vital for the normal balance and function of oxidative phosphorylation complex enzymes, and loss of ATAD3A reduces cellular ATP production partially through disruption of the oxidative phosphorylation complex. The highly damaged mitochondrial structure and disrupted mitochondrial content in the ATAD3A KO cells under FC overload can also be seen by TEM (Fig. 6*H*).

## Discussion

Even though ATAD3A has been reported to be involved in cholesterol transport, the function of ATAD3A in NAFLD remains unclear. Herein, we show the upregulation of ATAD3A in the progression of NAFLD in livers from humans and rats. Since it has been shown that cholesterol homeostasis disruption and FC overload contribute to NAFLD pathology, it is more relevant to investigate the function of ATAD3A under FC overload (3). We induced FC overload in Huh7 cells by treatment with cholesterol and 58035, an inhibitor of acetyl-CoA acetyltransferase 2. To understand the role of ATAD3A under FC overload, we established a stable Huh7 ATAD3A KO cell line using CRISPR-Cas9 gene editing. The results showed that ATAD3A KO induced FC and TG accumulation under FC overload conditions. Moreover, an increased number of small lipid droplets was observed in ATAD3A KO cells compared to WT Huh7 cells under control and FC overload conditions. In NAFLD, the total amount of lipid droplets/TG/neutral lipids is used to diagnose and characterize the disease (liver fats > 5% of liver weight). The finding that following the loss of ATAD3A, there is an increase in TG levels in liver cells even without treatment suggests that loss of ATAD3A may promote NAFLD initiation or development. In NAFLD patients, both microvesicular steatosis (accumulation of small fat droplets with preserved cellular architecture) and macrovesicular steatosis (the formation of larger droplets that displaces the nucleus) can be present in the liver (25).

Loss of ATAD3A upregulated LC3-II and p62 under FC overload in Huh7 cells and HLCM-derived human hepatocytes and downregulated PINK1 expression, suggesting ATAD3A KO under FC overload blocks autophagosome degradation and mitophagy. Furthermore, loss of ATAD3A impaired mitochondrial basal respiration and ATP production under FC overload in Huh7 cells.

Given that p-mTOR is upregulated in NASH-related cirrhosis (26) and mTOR is regulated by ATAD3A in cow epithelial cells (17), we expected to see upregulation of ATAD3A during the progression of NAFLD. Indeed, our results showed that in the rat NASH model and NAFLD human liver samples, ATAD3A levels were substantially increased.

In Huh7 cells, we found that after the loss of ATAD3A, mitochondria became dilated, and cristae were disrupted, which is consistent with previous findings (12). The altered structure of mitochondria and cristae after the loss of ATAD3A indicates potential changes in mitochondrial function. Subsequent functional analysis of mitochondria using the Seahorse assay uncovered impaired mitochondrial basal respiration and reduced ATP production after deleting ATAD3A, confirming mitochondrial dysfunction. Taken together, these data suggest that ATAD3A is pivotal for the mitochondrial structure and energy supply.

Unexpectedly, even though the loss of ATAD3A induced significantly higher FC accumulation under FC overload, neither key enzymes in cholesterol synthesis nor genes involved in cholesterol metabolism showed significant changes. Loss of ATAD3A significantly upregulated HMGR mRNA expression under control conditions but did not impact its expression under FC overload, even though there was a decline in HMGR mRNA expression in ATAD3A KO cells under FC overload. HMGR protein levels were downregulated rather than upregulated to contribute to the FC increase after loss of ATAD3A under FC overload. Furthermore, LXRA expression was not significantly affected by the loss of ATAD3A under FC overload either, even though a declining trend was seen, suggesting that ATAD3A may impact cholesterol export from cells to affect the intracellular cholesterol pool.

It is known that ATAD3A mutation or silencing alters autophagic levels (27, 28). In this study, we showed that autophagosome degradation was blocked after loss of ATAD3A. To take a closer look, the PINK1-Parkin–mediated mitophagy was impaired after the loss of ATAD3A under both control and FC overload conditions, which together with the altered mitochondrial structure and impaired mitochondrial respiration promoted the accumulation of damaged mitochondria. Mitophagy is progressively impaired with the progression of NAFLD, and enhancement in PINK1-Parkin–mediated mitophagy has been shown to ameliorate liver steatosis (29), revealing the importance of effective mitophagy for the restraint of disease. Our data suggests that ATAD3A is pivotal for the PINK1-Parkin–mediated mitophagy and loss of ATAD3A almost halted the mitophagy, making it a promising therapeutic target for NAFLD.

The upregulation of PLIN2 after the loss of ATAD3A indicates that suppressed lipolysis contributes to the TG accumulation. PLIN2 protects neutral lipids from lipolysis when coated at the surface of LDs and is identified as a therapeutic target for NAFLD. It has been shown that loss of PLIN2 in hepatocytes partially protected Western diet-fed mice from steatosis and largely from NASH, whereas overexpression of PLIN2 elevates both TG and LDs (30, 31). The ability of ATAD3A to regulate PLIN2 further predestinates its inseparable relationship with NAFLD.

Autophagy has also been shown to regulate lipid homeostasis, but it is controversial whether autophagy depletes or replenishes TG storage. Some studies have shown that autophagy reduces cellular lipids *via* degradation of LDs. In contrast, it has also been proposed that elevated autophagy may also contribute to the TG storage by degradation of cellular materials to provide FA for TG synthesis in hepatocytes (32, 33). In this study, FC overload dramatically induced autophagy and depleted TG stores in hepatocytes. However, ATAD3A KO blocked late-stage autophagy and accumulated more TG in the cells, suggesting that autophagy blockade may also contribute to TG accumulation after ATAD3A deletion.

**Figure 7:**
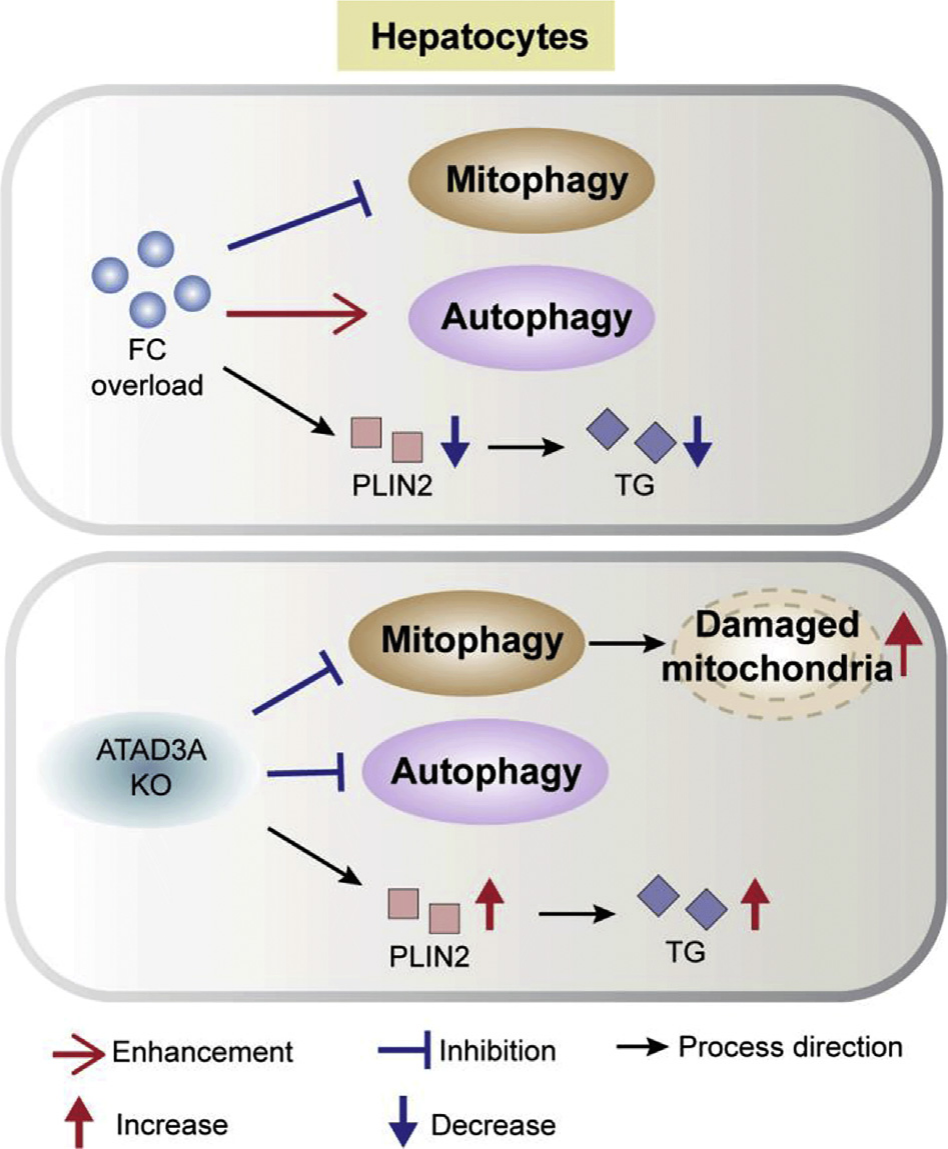
**Schematic representation of the influence of FC and loss of ATAD3A on autophagy, mitophagy, and lipids.** FC overload induces autophagy but blocks mitophagy and reduces PLIN2 expression and TG amount in cells. The loss of ATAD3A suppresses both autophagy and mitophagy, upregulates PLIN2, and accumulates TG in cells under normal conditions or when FC is overloaded. Collectively, ATAD3A deletion contributes to NAFLD through TG accumulation and compromised mitochondrial quality control in hepatocytes. FC, free cholesterol; PLIN2: perilipin 2; TG, triglyceride.

In conclusion, the loss of ATAD3A triggered accumulated FC and TG, blocked autophagosome degradation and effective degradation of damaged mitochondria with impaired mitochondrial respiration, and induced significantly higher liver cell death when challenged with FC overload, revealing the essential role of ATAD3A in NAFLD through the regulation of lipid homeostasis and mitochondrial health. A schematic representation of these events is shown in Figure 7. Admittedly, our *in vitro* lipid accumulation model cannot faithfully mirror the metabolic dynamics observed during the progression of NAFLD *in vivo*, which involves interactive signaling between different liver cell subtypes, such as hepatic stellate cells, Kupffer cells, and endothelial cells. Ultimately, *in vivo* animal studies are needed to confirm the *in vitro* observations. Nevertheless, our study proposes a pivotal role for ATAD3A in NAFLD progression and provides fundamental data to support the proposition that ATAD3A may be a potential target for NAFLD therapies.

## Experimental procedures

### Standard protocol approvals and patient consent

The NAFLD patient study was done in accordance with the guidelines approved by McGill University Health Centre Institutional Review Board. Prior written informed consent was obtained from all subjects who participated in the study (protocol: SDR-11-669). The studies in this work abide by the Declaration of Helsinki principles. A detailed description of the human liver samples used was recently published and is summarized in Table S1 (18).

### Experimental animals

For MCD diet studies, male rats aged 12 weeks old were fed either regular rat chow or a MCD diet (MP Biomedicals, #960439; n = 3) for 8 weeks. The rats were divided into 2 groups (chow and MCD n = 3). The body weight was recorded every week. After 2 months, the rats were harvested, and fresh liver tissue samples were placed into micro-Eppendorf tubes and stored in −80 °C for protein measurement. The rats were bred and maintained in accordance with protocols approved by the IACUC of the University of Southern California.

### Cell culture

Huh7 cells were purchased from JCRB Cell Bank (originally isolated from a liver tumor from a 57-year-old Japanese male) and maintained in Dulbecco's Modified Eagle Medium (DMEM) (#11995-065, Gibco) supplemented with 10% of fetal bovine serum (FBS) (#12306C, Millipore Sigma) and 1% of Penicillin-Streptomycin (#15140-122, Gibco) at 37 °C and 5% CO_2_ incubator. The medium was replaced every 3 days if not otherwise stated.

Human hepatocytes were cultured in DMEM (#11995-065, Gibco) supplemented with 20 mM Hepes (#15630-080, ThermoFisher), 10% of FBS (#12306C, Millipore Sigma), 1% of Penicillin-Streptomycin (#15140-122, Gibco), 15 μg/ml L-Proline (#219472825, MP Biomedicals), 0.25 μg/ml insulin (#I1882, Millipore Sigma), 50 nM dexamethasone (#D8893, Millipore Sigma), 5 ng/ml epidermal growth factor (#E9644, Millipore Sigma), 0.1 mM L-ascorbic acid 2-phosphate (Asc-2P) (#013-12061, FUJIFILM Wako Chemicals), 2% DMSO (#D2650, Millipore Sigma) at 37 °C and 5% CO_2_ incubator after isolation. The medium was replaced every 3 days within 1 week after isolation. Then the medium was replaced to DMEM (#11995-065, Gibco) supplemented with 10% of FBS (#12306C, Millipore Sigma) and 1% of Penicillin-Streptomycin (#15140-122, Gibco) for continued culture until experimental treatments.

### CRISPR-Cas9 KO in Huh7 cells

ATAD3A CRISPR KO Vector ALL In One (set of 3 targets) (#125971110595, abm) encoding sgRNA targeting ATAD3A exon 7 sequence 5′-CGAGCAGATCCGCCTGAAGG-3′ and Cas9 was transfected into Huh7 cells followed by 2 μg/ml puromycin (#A1113803, ThermoFisher) selection. Transfected Huh7 cells were then redistributed onto a 100-mm culture dish to allow the formation of single cell colonies. After culturing for one month, single cell colonies were picked up under the microscope and transferred to 6-well plates. When single cell colonies reached 80% confluency, protein was extracted and subjected to immunoblotting to detect the loss of ATAD3A.

### Transient ATAD3A overexpression in Huh7 cells

Customized lentiviral vector expressing ATAD3A isoform 2 and control vector were purchased from VectorBuilder. Lipofectamine 3000 Transfection Reagent (#L3000015, ThermoFisher) was used for transfections following manufacturer’s instructions. Briefly, Huh7 cells were seeded onto 6-well plates at a density of 0.4 × 10^6^/ml. One hour before transfection, the medium was replaced with fresh complete medium. A liquid mixture of lipofectamine 3000 reagent and lentiviral vector was added to cells and incubated for 24 h. Then the medium was replaced with DMEM supplemented with 10% of Lipoprotein Depleted Fetal Bovine Serum (#880100-5, Kalen Biomedical, LLC) overnight, followed by the treatment with cholesterol and 58035 for 24 h. The cell pellet was then collected and subjected to immunoblotting.

### Cholesterol and 58035 treatment

Huh7 cells or human hepatocytes were seeded at a density of 0.4 × 10^6^/ml, 0.2 × 10^6^/ml, or 0.1 × 10^6^/ml in 6-well plates, 12-well, and 24-well plates or 96-well plates, respectively, with DMEM supplemented with 10% of Lipoprotein Depleted Fetal Bovine Serum (#880100-5, Kalen Biomedical, LLC) and incubated overnight. Cholesterol–methyl-β-cyclodextrin (#C4951-30MG, Millipore Sigma) and Sandoz 58035 (#S9318-5MG, Millipore Sigma) were added to the medium to a final concentration of 100 μM and 10 μg/ml, respectively, and incubated for 24 h. 0.01% DMSO (#WN182-10Ml, VWR) was added to cells as the control. The pretreatment time was 1 h for both 3-MA (#M9281, Millipore Sigma) (2 mM) and bafilomycin A1 (#SML1661, Millipore Sigma) (100 nM).

### Confocal microscopy

Huh7 cells were seeded on coverslips in DMEM (#11995-065, Gibco) supplemented with 10% of Lipoprotein Depleted Fetal Bovine Serum (#880100-5, Kalen Biomedical, LLC) overnight prior to treatment with 100 μM cholesterol and 10 μg/ml 58035 for 24 h. Cells were then incubated with phenol red-free DMEM (#31053-028, Gibco) containing 100 nM MitoTracker Deep Red FM (#M22426, ThermoFisher) for 30 min at 37 °C. After washed with PBS, cells were immobilized with 4% paraformaldehyde (#R37814, ThermoFisher) for 10 min, incubated with 0.1% of Triton-X (#PI85111, ThermoFisher) for 10 min, and blocked with PBS (#sc-362183, ChemCruz) containing 5% of donkey serum (#D9663-10Ml, Millipore Sigma) and 0.5% of bovine serum albumin (BSA) (#BAH65, Equitech-Bio, Inc) for 30 min. Cells were then incubated with primary antibody against ATAD3A (#H00055210-D01, Abnova) with a dilution of 1:400 at 4 °C overnight, followed by the addition of the Donkey anti-Rabbit IgG (H + L) Highly Cross-Adsorbed Secondary antibody, Alexa Fluor 568 (#A10042, ThermoFisher) for 1 h. Cells were stained with Duolink *In Situ* Mounting Medium with DAPI (#DUO82040, Millipore Sigma). Images were taken with a ZEISS LSM 880 Confocal Microscope. The colocalization analysis was performed using ImageJ v1.52a.

### Human hepatocyte isolation from HLCM

Human hepatocytes were isolated from HLCM as described previously. In brief, HLCM were produced by an injection of commercially available cryopreserved primary human hepatocytes (PHH; Lot: JFC, 1-year old, Caucasian male, BioIVT Westbury) into the spleen of severe combined immunodeficiency mice with albumin enhancer/promoter driven cDNA-urokinase type plasminogen activator transgene expression as previously described (34). The use of PHH for the production of HLCM has been approved by Utilization of Human Tissue Ethical Committee of PhoenixBio Co, Ltd (0031). After 13 to 27 weeks post PHH injection, HLCM with high replacement rates (>90%, estimated by blood human albumin levels, which is highly correlated with histological replacement index) (35) were subjected to human hepatocyte isolation. Human hepatocytes were isolated by a two-step collagenase perfusion method as described previously (36).

### RNAi treatment of human hepatocytes isolated from HLCM

RNAi of ATAD3A was conducted using Lipofectamine RNAiMAX Transfection Reagent (#13778150, Invitrogen) following the manufacturer's instructions. Briefly, human hepatocytes were grown in culture medium overnight before the day of transfection. One hour before transfection, the medium was replaced with fresh complete medium. The mixture of Lipofectamine RNAiMAX Reagent and human ATAD3A siRNA (#NM_018188 siRNA 1, Millipore Sigma) was then added and incubated for 24 h. The MISSION siRNA Universal Negative Control #1 (#SIC001-5X1NMOL, Millipore Sigma) was used as control siRNA.

### Immunoblotting

Cells were harvested at 5000 rpm for 5 min and washed with PBS. Lysis buffer (#sc-24948A, ChemCruz) was added to the cell pellet, and cell extracts were incubated on ice for 30 min. Cell extracts were then centrifuged at 17,200*g* at 4 °C for 30 min to remove cell debris. Supernatants were taken out, and protein concentrations were determined using Pierce BCA Protein Assay Kit (#23225, ThermoFisher). Loading buffer (#J61337-AC, Alfa Aesar) was added to cell extracts, and the mix was heated to 95 °C for 10 min in a digital heatblock (VWR). Samples were loaded onto a protein gel (#4568095, Bio-Rad) and run under 100 V for 1 h. The protein in the gel was transferred to polyvinylidene difluoride membrane (#ISEQ00010, Millipore Sigma) under 80 V for 2 h. The membrane was blocked with 5% of BSA for 30 min and incubated with primary antibodies against HMGR (#SAB4200529, Millipore Sigma), LXRA (#ab176323, abcam), SREBP1c (#66875-1-Ig, proteintech), FAS (#3180, cell signaling), PLIN2 (#A6276, ABclonal), PPARA (#66826-1-Ig, proteintech), CPT1A (#ab128568, abcam), ATAD3A ((#H00055210-D01, Abnova), LC3-II (#2775, cell signaling), p62 (#5114, cell signaling), PINK1 (#ab23707, abcam), Parkin (#14060-1-AP, proteintech), COX IV (#ab14744, abcam), TOM20 (#ab56783, abcam), Total OXPHOS (#ab110411, abcam), GAPDH (#60004-1-Ig, proteintech), or alpha-Tubulin (#T9026-.2Ml, Millipore Sigma) at 4 °C overnight. After washing with PBST (PBS with 0.1% Tween 20 (#8221840500, Millipore Sigma)), 5% of BSA with Goat anti-Rabbit HRP Secondary antibody (#926-80011, LI-COR) or Goat anti-Mouse HRP Secondary antibody (#926-80010, LI-COR) was added to the membrane and incubated at RT for 1 h. The images were taken by Azure c600 biosystem (Azure Biosystems). Following immunoblot analysis with a specific antibody, membranes were stripped of the antibodies with Stripping Buffer (#21063, ThermoFisher) and reblotted with an antibody targeting an internal control, for example, GAPDH or alpha-Tubulin. In search of more than one antigen of different molecular weights, separated and blotted on the same membrane, the membrane was cut, and each strip blotted with antibodies specific to antigens and internal control, for example, GAPDH or alpha-Tubulin.

### Transmission electron microscopy

1 x 10^6^ cells were harvested, pelleted, and fixed in 1/2 strength Karnovsky solution, containing 2.5% glutaraldehyde and 2% paraformaldehyde in PBS, overnight at 4 °C. The cell pellet was rinsed in a 0.1 M cacodylate buffer and postfixed in 2% aqueous OsO4/0.2 M cacodylate buffer for 2 h at 4 °C, followed by several rinses with 0.1 M cacodylate buffer. An EM block was stained with saturated uranyl acetate overnight at 4 °C. After rinsing with 0.1 M sodium acetate buffer, the cells were dehydrated through a gradient series of 30%–100% ethanol, placed in 100% propylene oxide, and infiltrated in a 1:1 mixture of propylene oxide:polybed 812 epoxy resin (#21844-1, Polysciences) overnight at room temperature. After several changes of 100% resin over 24 h, the pellet was embedded in Quetol 812 (#GN341, Nissin EM Co). Areas containing cells were block mounted and cut into 70-nm sections with a Leica EM UC6 Ultramicrotome (Leica, Germany). The sections were stained with uranyl acetate (saturated aqueous solution) and lead citrate and examined with a transmission electron microscope (H-7100, Hitachi).

### Cholesterol quantification

The cholesterol was quantified using Cholesterol Quantitation Kit (#MAK043, Millipore Sigma) following the manufacturer's instructions. Briefly, 1 × 10^6^ cells were pelleted at 5000 rpm for 5 min and washed with PBS. Two hundred microliters of chloroform:isopropanol:IGEPAL CA-630 (7:11:0.1) were added, and samples were homogenized at an oscillation frequency of 60/s for 1 min in TissueLyser II (Qiagen) to extract the cholesterol. Cell lysates were centrifuged at 17,200*g* for 30 min to remove insoluble material. The supernatant was taken out and air dried at 50 °C in a speed vacuum for 1 h. The dried lipids were redissolved in 250 μl of assay buffer. For the assay, 50 μl of each sample along with 50 μl reaction mix were set up in duplicate using 96 well plates and incubated at 37 °C for 1 h. The absorbance at wavelength of 570 was detected by a multimode plate reader (PerkinElmer VICTOR X5). The amount of cholesterol was normalized to the protein concentration determined by Pierce BCA Protein Assay Kit (#23225, ThermoFisher).

### Filipin staining

Huh7 cells were seeded on coverslips in DMEM (#11995-065, Gibco) supplemented with 10% of Lipoprotein Depleted Fetal Bovine Serum (#880100-5, Kalen Biomedical, LLC) overnight prior to treatment with 100 μM cholesterol and 10 μg/ml 58035 for 24 h. Huh7 cells were washed with PBS and immobilized by adding 4% (wt/vol) paraformaldehyde for 10 min. Cells were then washed three times with PBS and incubated with 0.1% of Triton-X for 10 min. The cells were then washed three times again in PBS and blocked with PBS containing 5% of donkey serum and 0.5% BSA for 30 min. After washing three times with PBS , cells were incubated with 0.05 mg/ml Filipin (#F4767-1MG, Millipore Sigma) and primary antibody against LaminB1 (#ab16048, abcam) at a dilution of 1:400 at a room temperature for 2 h. Cells were again washed three times with PBS and incubated with Goat anti-Rabbit IgG (H + L) Cross-Adsorbed Secondary antibody, Alexa Fluor 488 (#A11008, ThermoFisher) for 1 h. After another three washes with PBS, the cells were mounted with Fluoromount-G Slide Mounting Medium (#17984-25, Electron Microscopy Sciences). The images were visualized and captured with fluorescent microscopy (REVOLVE, Echo).

### qPCR

RNA was isolated using Quick-RNA Miniprep Plus Kit (#R1058, ZYMO RESEARCH). RNA was reverse transcribed to cDNA using PrimeScript RT Master Mix (Perfect Real Time) (#RR036A, TaKaRa), following the manufacturer’s instructions, by Mastercycler nexus X2 (Eppendorf). The qPCR was conducted by Biotek. The internal control is human ribosomal protein S18 (Rps18). The expression was normalized to S18 mRNA. The primer sequences were as follows: HMGR-F: 5′-AGTGAGATCTGGATCCAA-3'; HMGR-R: 5′-GATGGGAGGCCACAAAGAGG-3'; LXRA-F: 5′-TCTGGACAGGAAACTGCACC-3'; LXRA-R: 5′-CCGCAGAGTCAGGAGGAATG-3'; SREBP1C-F: 5′-TGCTGCAGGAGTCTGAGAGA-3'; SREBP1C-R: 5′-CTGGACCAGACTCTGCCTTG-3'; FAS-F: 5′-GCAAGCTGAAGGACCTGTCT-3'; FAS-R: 5′-AATCTGGGTTGATGCCTCCG-3'; PLIN2-F: 5′-TGATGGCAGGCGACATCTAC-3'; PLIN2-R: 5′-AAAGGGACCTACCAGCCAGT-3'; PPARA-F: 5′-TGGGAAGGCAGCGTTGATTA-3'; PPARA-R: 5′-CTGGCAGTTCCAGTCCAGAT-3'; CPT1A-F: 5′-GTTCTCTTGCCCTGAGACGG-3'; CPT1A-R: 5′-TTTCCAGCCCAGCACATGAA-3'; p62-F: 5′-AGCGTCAGGAAGGTGCCATT-3'; p62-R: 5′-TTCTCAAGCCCCATGTTGCAC-3'; S18-F: 5′-ATTAAGGGTGTGGGCCGAAG-3’; and S18-R: 5′-TGGCTAGGACCTGGCTGTAT-3’.

### TG quantification

TGs were quantified using Triglyceride Quantification Kit (#MAK266, Millipore Sigma) following the manufacturer's instructions with minor modifications. Briefly, 1 × 10^6^ cells were harvested and homogenized in 500 μl solution of 5% Nonidet P 40 Substitute (#492016-500 ml, Millipore Sigma). Samples were then heated to 100 °C in a digital heatblock (VWR) for 2 to 5 min until the Nonidet P 40 becomes cloudy, followed by cooling temperature to room temperature. Samples were centrifuged at 17, 200*g* for 5 min to remove insoluble material at room temperature. The supernatant was collected followed by the addition of lipase and master reaction mix in the kit. The absorbance at 570 nm was measured by Synergy H1 multiplate reader (BioTek). The amount of TG was normalized by the protein concentration determined by Pierce BCA Protein Assay Kit (#23225, ThermoFisher).

### LDs staining with Nile red

Huh7 cells (0.2 × 10^6^ cells/ml) were seeded on coverslips in DMEM (#11995-065, Gibco) supplemented with 10% of Lipoprotein Depleted Fetal Bovine Serum (#880100-5, Kalen Biomedical, LLC) overnight. Cells were treated with 100 μM cholesterol and 10 μg/ml 58035 for 24 h and fixed with 4% (wt/vol) paraformaldehyde for 10 min and washed with PBS for three times. Glycine (#G7126, Millipore Sigma; 50 mM) was then added to quench the cells for 10 min at room temperature. After washing with PBS for three times, the cells were incubated with 10 μg/ml Nile red (#19123-10MG, Millipore, Sigma) at room temperature for 10 min. After completion of staining, the Nile red was removed, and cells were washed with PBS. Cells were then stained with Duolink *In Situ* Mounting Medium with DAPI (#DUO82040, Millipore Sigma). Images were visualized and captured by fluorescent microscopy (REVOLVE, Echo).

### MitoTracker staining

Huh7 cells were seeded on coverslips in DMEM (#11995-065, Gibco) supplemented with 10% of Lipoprotein Depleted Fetal Bovine Serum (#880100-5, Kalen Biomedical, LLC) overnight before treatment with 100 μM cholesterol and 10 μg/ml 58035 for 24 h. Cells were then washed with PBS and incubated with phenol red-free DMEM (#31053-028, Gibco) containing 100 nM MitoTracker Green FM (#M7514, ThermoFisher) for 30 min to allow for the internalization of MitoTracker into cells. Cells were washed with PBS three times and mounted with Fluoromount-G Slide Mounting Medium (#17984-25, Electron Microscopy Sciences). The images were visualized and captured with fluorescent microscopy (REVOLVE, Echo).

### MTT cell proliferation assay

The MTT assay was conducted using cell proliferation kit I (MTT) (#11465007001, Roche), following the manufacturer's instructions. Briefly, cells were seeded at a density of 10,000 cells/well in a 96-well plate followed by 24-h incubation with treatments. After incubation, 10 μl of MTT labeling reagent was added to each well and incubated for 4 h in 37 °C. Hundred microliters of solubilizing solution was subsequently added and incubated overnight in 37 °C incubator. The absorption at 595 nm was measured by a multimode plate reader (PerkinElmer VICTOR X5).

### Cell number count in human hepatocytes isolated from HLCM

Human hepatocytes from HLCM were isolated and seeded on coverslips followed by the RNAi for ATAD3A using Lipofectamine RNAiMAX Transfection Reagent (#13778150, Invitrogen) following the manufacturer's instructions for 24 h. Cells were then washed with PBS and incubated in DMEM (#11995-065, Gibco) supplemented with 10% of Lipoprotein Depleted Fetal Bovine Serum (#880100-5, Kalen Biomedical, LLC) overnight before treatment with 100 μM cholesterol and 10 μg/ml 58035 for 24 h. Cells were then washed with PBS, and the nuclei were stained with DAPI (#DUO82040, Millipore Sigma). Cell number was counted in three randomly selected areas for each sample under the microscope (REVOLVE, Echo).

### Seahorse assay for oxygen consumption rate

The Seahorse assay was conducted using Agilent Seahorse XF Cell Mito Stress Test Kit (#103015-100, Agilent) and Seahorse XFe96 Analyzer following the manufacturer's instructions. Briefly, 10,000 cells were seeded onto the Seahorse XF Cell Culture Microplate (#101085-004, Agilent) two days before assaying. One day before the assay, cells were treated with cholesterol and 58035 for 24 h while the microplate cartridge was hydrated by 200 μl Molecular Biology Grade Water (#46-000-CI, Corning) overnight at 37 °C. At the day of assay, the water in microplate cartridge was replaced by prewarmed XF Calibrant (#100840-000, Agilent) followed by incubation in 37 °C non-CO_2_ incubator for 1 h. Then, 20 μl of 100 μM oligomycin, 22 μl of 100 μl 100 μM FCCP, and 25 μl of 50 μM Rotenone/AA was added to the port A, B, C of the sensor cartridge, respectively. For the cell culture microplate, the medium was replaced by Agilent Seahorse XF DMEM Medium (#103575-100, Agilent) supplemented with 1 mM pyruvate (#103578-100, Agilent), 2 mM glutamine (#103579-100, Agilent), and 10 mM glucose (#103577-100, Agilent), followed by 1 h incubation in a 37 °C non-CO_2_ incubator. An XF cell mito stress-test template was used to measure oxygen consumption rate of cells. The data was normalized to protein concentration in Wave 2.6.1 software.

### Statistical analysis

Data analyses were performed with GraphPad Prism 8.0.2 software. Data with two groups and six groups were analyzed by unpaired two-tailed student's t test and one-way ANOVA, respectively. Total cholesterol and FC were analyzed using two-way ANOVA. p-values < 0.05 were considered statistically significant.

## Data availability

The authors confirm that the data supporting the findings of this study are available within the article and its supplementary materials.

## Supporting information

This article contains supporting information.

## Conflict of interest

The authors declare that they have no conflicts of interest with the contents of this article.

## Supporting information

ATAD3A-liver-S Data-title page-Figs-Table-JBC1-Fin.Pdf
