# Supplementary material for: Loss of mitochondrial ATPase ATAD3A contributes to nonalcoholic fatty liver disease through accumulation of lipids and damaged mitochondria
Source: J Biol Chem. 2022 May 2;298(6):102008. doi: 10.1016/j.jbc.2022.102008 (PMC9157002; doi:10.1016/j.jbc.2022.102008)
Supplement: ATAD3A-liver-S Data-title page-Figs-Table-JBC1-Fin.Pdf [file mmc1.pdf]

Supplemental Data

Loss of mitochondrial ATPase ATAD3A contributes to nonalcoholic fatty liver disease through accumulation of lipids and damaged mitochondria

Liting Chen,[1] Yuchang Li,[1] Chantal Sottas,[1] Anthoula Lazaris,[2,3] Stephanie K Petrillo,[2,3] Peter Metrakos,[2,3] Yuji Ishida,[4,5] Takeshi Saito,[4,6] Lu Li,[1] Samuel Garza,[1] Vassilios Papadopoulos[1]

[1]Department of Pharmacology and Pharmaceutical Sciences, School of Pharmacy, University of Southern California, Los Angeles, CA 90089, USA
[2]Research Institute of the McGill University Health Center, Montreal, Quebec H4A 3J1, Canada
[3]Department of Surgery, McGill University, Montreal, Quebec H3G 1A4, Canada
[4]Department of Medicine, Division of Gastrointestinal and Liver Diseases, Keck School of Medicine, University of Southern California, Los Angeles, CA 90089, USA
[5]Research & Development Department, PhoenixBio, Co., Ltd, Higashi-Hiroshima, Hiroshima, Japan
[6]University of Southern California Research Center for Liver Diseases, Los Angeles, CA 90089, USA

Figure S1. ATAD3A KO increases the number of small size LDs. A, B. Nile red staining and quantification for Huh7 WT or KO treated as indicated. Scale bar, 130 μm. Chol, cholesterol; WT, wild type; KO, knockout; LDs, lipid droplets.

Figure S2. ATAD3A overexpression promotes autophagic degradation under FC overload. WB for ATAD3A, LC3-I, LC3-II and p62 in Huh7 cells transfected with plasmid carrying ATAD3A mRNA variant 2 or control plasmid. FC, free cholesterol, OE, overexpression.

Table S1. NAFLD activity score (NAS) for normal human or patient liver samples. (Related to main Figure 1).

Figure S1

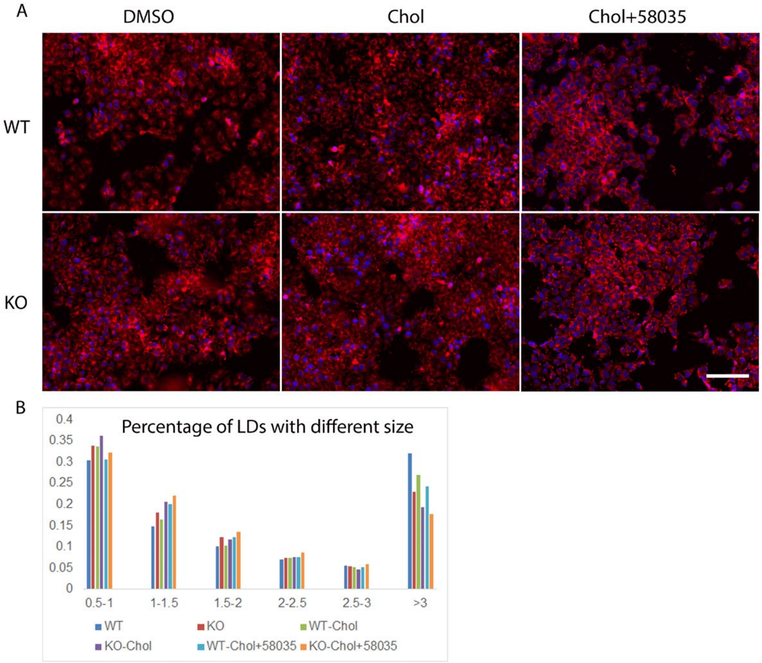
A
DMSO
Chol
Chol+58035
WT
KO
B
Percentage of LDs with different size
0.4
0.35
0.3
0.25
0.2
0.15
0.1
0.05
0
0.5-1
1-1.5
1.5-2
2-2.5
2.5-3
>3
WT
KO
WT-Chol
KO-Chol
WT-Chol+58035
KO-Chol+58035

Figure S2

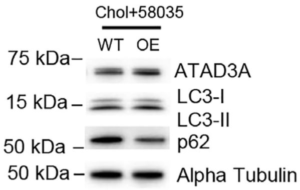
Chol+58035
WT
OE
75 kDa
ATAD3A
15 kDa
LC3-I
LC3-II
50 kDa
p62
50 kDa
Alpha Tubulin

Table S1. NAFLD activity score (NAS) for the normal human or patient liver samples. (Related to main Figures 1)

	Sex	Age	Type of Procedure	Diagnosis	Steatosis	Lobular Inflammation	Hepatocyte Ballooning	Fibrosis score	% Micro steatosis	% Macro steatosis	% Total steatosis	NA score	Grading
Normal	Female	52	Resection	Biliary cystadenoma	0	0	1	1A	0	0	0	2	LOW
NAFLD	Male	64	Resection	CRCLM	2	1	0	1A	15	30	45	4	NAFLD
NASH	Female	65	Resection	CRCLM	2	2	1	1A	10	30	40	6	NASH
Cirrhosis	Female	65	Resection	Benign	0	3	1	3	0	0	0	7	Cirrhotic

Note: CRCLM: colorectal cancer liver metastases.
